# The three-tube method via precise interventional placement for esophagojejunal anastomotic fistula after gastrectomy: a single-center experience

**DOI:** 10.1186/s12957-023-03105-7

**Published:** 2023-08-01

**Authors:** Xiaolong Ding, Chenchen Zhang, Xiaobing Li, Tao Liu, Yaozhen Ma, Meipan Yin, Chunxia Li, Gang Zhou, Gang Wu

**Affiliations:** 1 Department of Interventional Radiology, The First Affiliated Hospital of Zhengzhou University, Zhengzhou, 450052 China; 2 Department of Gastrointestinal Surgery, The First Affiliated Hospital of Zhengzhou University, Zhengzhou, 450052 China

**Keywords:** Gastrectomy, Anastomotic fistula, Complication, Interventional radiology

## Abstract

**Background:**

Esophagojejunal anastomotic leakage is a serious complication after total gastrectomy. This study evaluated the safety and efficacy of transnasal placement of drainage catheter, jejunal decompression tube, and jejunal nutrition tube under fluoroscopy for treatment of esophagojejunal anastomotic fistula after gastrectomy in gastric cancer patients.

**Methods:**

This is retrospective review of patients with esophagojejunal anastomotic fistula treated with transnasal placement of abscess drainage catheter, decompression tube, and jejunal nutrition tube under fluoroscopy. Fistula healing time, patient survival, and Eastern Cooperative Oncology Group (ECOG) performance status before and after treatment were evaluated.

**Results:**

Sixty-four patients were included in the study. Insertion of the transnasal abscess drainage catheter, decompression tube, and jejunal nutrition tube was successful on the first attempt in all patients, while 35 patients received transnasal abscess drainage, 13 received percutaneous abscess drainage, and 16 received transnasal drainage plus percutaneous abscess drainage. Immediately after placement of the tube, the mean volume of drainage was 180 mL (range, 10–850 mL); the amount steadily decreased from then on. The clinical success rate was 84.3% (54/64). Median time to fistula healing was 58 days (range, 7–357 days).

**Conclusions:**

Transnasal insertion of transnasal abscess drainage catheter, jejunal decompression tube, and jejunal nutrition tube under fluoroscopy appears to be a simple, minimally invasive, effective, and safe method for treating esophagojejunal anastomotic fistula after gastrectomy.

## Background

Esophagojejunal anastomotic fistula develops in 2–27% of patients receiving total gastrectomy and esophagojejunostomy for treatment of resectable gastric cancer and can seriously impact quality of life and prognosis; the mortality rate exceeds 50% [1–5]. Early clinical manifestations of the fistula are an increase in the amount of drainage, pus-like changes in the drainage fluid, or poor drainage and intermittent fever. Without intervention, abscesses may form and lead to persistent high fever and abdominal pain and complications such as peritonitis, mediastinitis, chest infection, multiple organ failure, sepsis, respiratory failure, and death. The presence of esophagojejunal anastomotic fistula can be confirmed by the methylene blue test, esophagography, or endoscopy [3].

Currently, there is no consensus on the best treatment for esophagojejunal anastomotic fistula. Treatments include fasting with nil by mouth and complete parenteral nutritional support, mucosal protective agents, gastrointestinal decompression, percutaneous thoracic drainage tube placement, surgical drainage tube placement, surgical repair, esophageal stents, titanium clips, and endoluminal vacuum treatment (EVT) [1–8].

Interventional treatment of mediastinal abscess after spontaneous esophageal rupture and esophageal cancer has been shown to be safe and effective [9, 10]. However, there are no reports in literature of interventional treatment of esophagojejunal anastomotic fistula. At our hospital, we have been treating esophagojejunal anastomotic fistula by transnasal placement of abscess drainage catheter, decompression tube, and jejunal nutrition tube under fluoroscopy. The aim of this study is to report our 10-year experience with the use of this interventional procedure.

## Materials and methods

The data of patients with esophagojejunal anastomotic fistula treated by the three-tube method at our hospital between June 2012 and September 2022 were retrospectively reviewed. Patients were eligible for inclusion in this study if they (1) had undergone total gastrectomy and esophagojejunostomy for treatment of gastric cancer; (2) had esophagojejunal anastomotic fistula confirmed by the methylene blue test, gastrointestinal angiography, or computed tomography (CT); and (3) had received transnasal placement of abscess drainage catheter, decompression tube, and jejunal nutrition tube under fluoroscopy. Patients were excluded if (1) they had esophagogastric anastomotic fistula after non-total gastrectomy, (2) they had duodenal stump fistula, or (3) the esophagojejunal anastomotic fistula had healed after conservative treatment, surgical treatment, or endoscopic treatment.

The following data were collected for analysis: sex, age, and comorbidities; history of gastric cancer-related chemotherapy; pre-procedure American Society of Anesthesiologists (ASA) score and procedural severity score (PSS); pre- and post-procedure laboratory test results and Eastern Cooperative Oncology Group (ECOG) performance status; time from surgical resection to diagnosis of fistula; fistula size, location, and classification; time from diagnosis of fistula to interventional treatment; extent of abscess cavity; volume of drainage from abscess cavity; pus culture results during treatment and follow-up; morphological changes in fistula and abscess cavity; complications (infection, bleeding, shock, and so on); laboratory and imaging examination results; ECOG performance status at last follow-up; time of healing of fistula or death; and cause of death.

The institutional ethics committee approved this study (2022-KY-0024–001). All patients signed written informed consent.

### Preoperative preparation

**Fig. 1:**
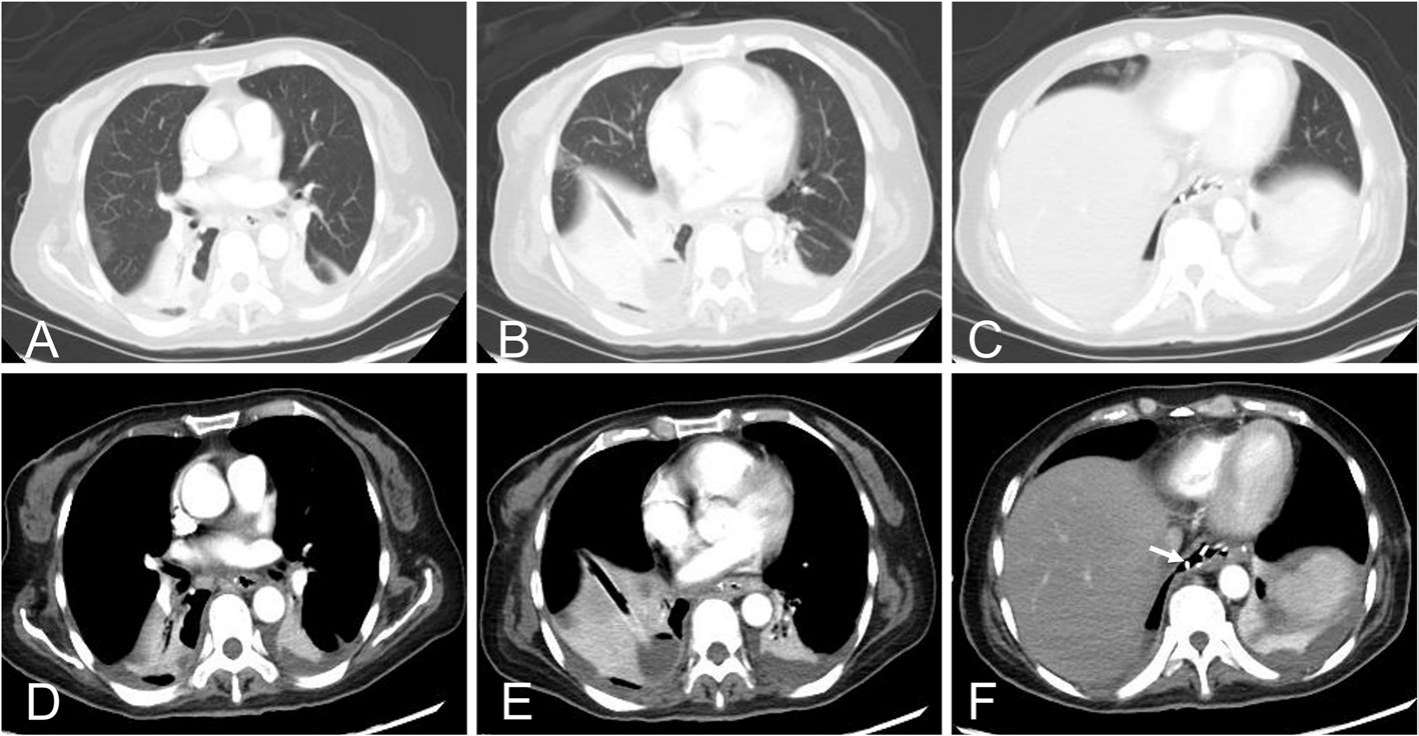
A 56-year-old female who underwent total gastrectomy and esophagojejunostomy for gastric cancer developed fever and chest tightness 4 days after surgery. CT examination revealed communication between the esophagojejunal anastomosis and the right thoracic cavity (white arrow), with inflammation, atelectasis of the lung, and fluid accumulation in the pleural space

Preoperatively, all patients underwent blood routine examination, liver and kidney function tests, serum electrolytes estimation, electrocardiogram, methylene blue test, upper gastrointestinal contrast study, and plain and enhanced chest CT scans (Fig. 1). Pre-procedure preparation was with fasting, gastric mucosal protective agents, parenteral nutrition, and prophylactic antibiotics. Patients with dyspnea or oxygen saturation < 90% received oxygen by nasal cannula and, if necessary, tracheal intubation and ventilator-assisted breathing.

### Procedure

**Fig. 2:**
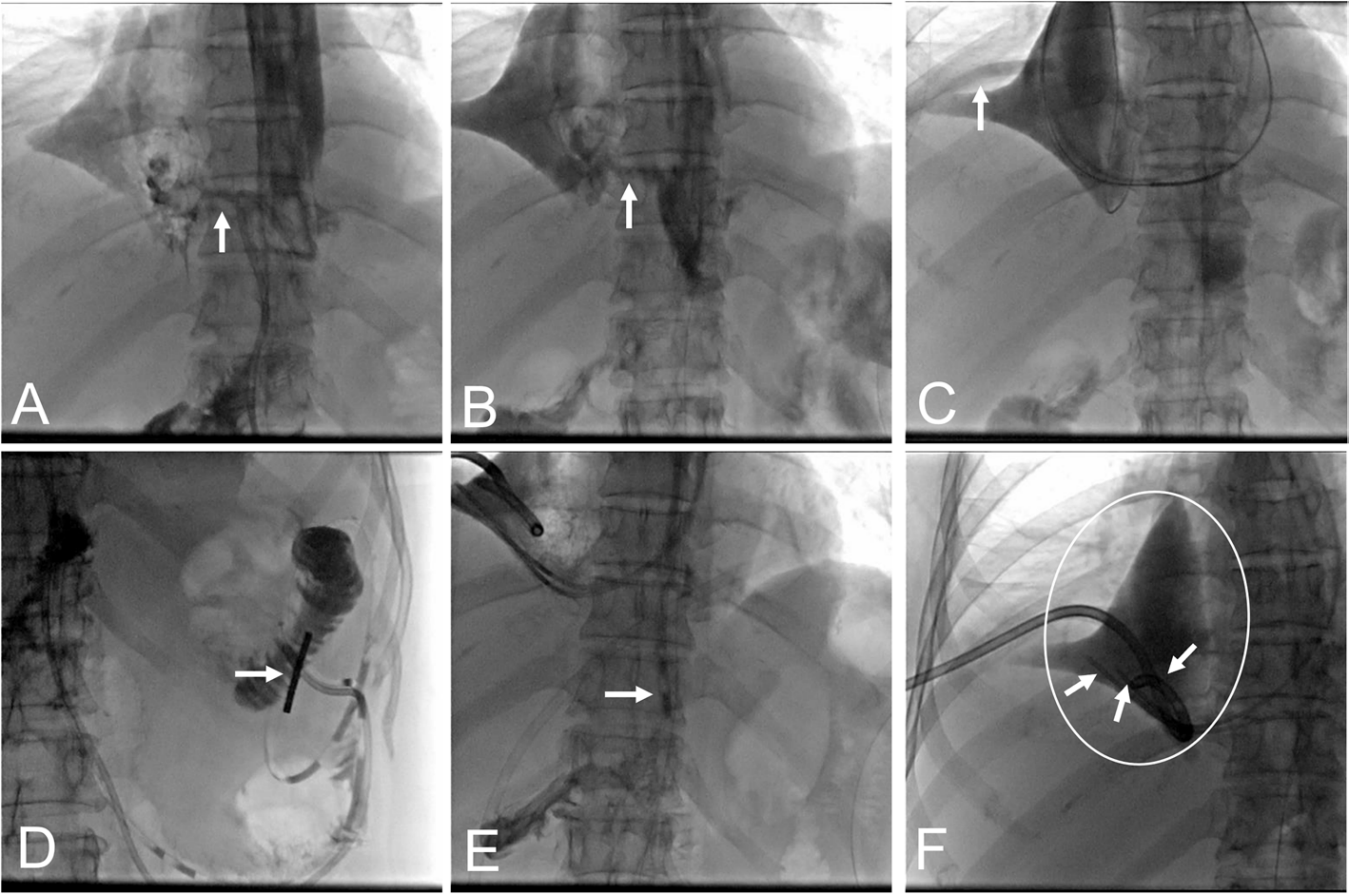
**A**–**B** Esophageal angiography shows contrast overflowing through the esophagojejunal anastomosis into the right chest cavity. **C** Under the guidance of the guide wire, two straight head lateral foramen catheters enter the right pus cavity through the fistula opening; the surgical drainage tubes can be seen in the pus cavity. **D** Under fluoroscopy, the tip of the nutrient tube is seen at the proximal end of the jejunum. **E** The side openings in the gastric tube spans the fistula opening. **F** The surgical drainage tube was replaced with a 12-F external drainage tube; the three pus drainage tubes are located in the pus cavity. The range and position of the pus cavity are marked with white circles

Esophagography was performed first. The nasal cavity, pharynx, and esophagus were anesthetized with oxybuprocaine hydrochloride gel 10 min after the esophagography. With the patient supine on the digital subtraction angiography table, ioversol was administered orally. Frontal, 45° left anterior oblique, and 45° right anterior oblique views of the esophagus were studied to determine the position and size of the esophagojejunal anastomotic leakage and the extent of spillage of contrast (Fig. 2A–B).

### Transnasal insertion of jejunal nutrition tube and decompression tube

Under fluoroscopy, a 0.035-inch hydrophilic membrane guide wire (Terumo Corporation, Japan) and a 5F vertebral artery catheter (Johnson & Johnson Cordis, USA) were passed through one nostril and advanced through the pharynx and esophagus, across the esophagojejunal anastomosis area into the upper jejunum. The catheter was withdrawn, and a jejunal nutrition tube was passed over the guide wire 40–50 cm into the jejunum. In the same way, a decompression tube was inserted through the other nostril into the jejunum 5–10 cm beyond the esophagojejunostomy (Fig. 2D–E).

### Transnasal insertion of abscess drainage tube

The drainage catheter and guide wire were introduced along the side of the jejunal decompression tube into the esophagus. Transcatheter esophagography was performed to display the location of the esophagojejunal anastomotic fistula, and the catheter and guide wire were then advanced into the abscess cavity through the fistula. The lowest pole of the abscess cavity was gently probed, and a 5F Performa® vessel catheter (Merit Medical, USA) was exchanged and advanced until the tip was at the lowest pole of the abscess cavity. If the abscess cavity was large, a 5F pigtail catheter (Merit Medical, USA) was inserted into the abscess cavity in the same way (Fig. 2C).

### Percutaneous abdominal drainage tube replacement

For patients with a percutaneous abscess drainage tube inserted during previous surgery, the tube was exchanged for a 10.2F or 12F external drainage tube, which was then connected to a negative-pressure (up to − 125 mmHg) suction system (Fig. 2F).

### Postoperative treatment and follow-up

Post procedure, the patients received fasting, jejunal nutrition, appropriate intravenous nutritional supplements, and continuous negative-pressure suction through the abscess drainage tube. Antibiotics were prescribed according to culture results. Normal-saline lavage was performed 1–2 times per day through the transnasal or percutaneous drainage tube.

**Fig. 3:**
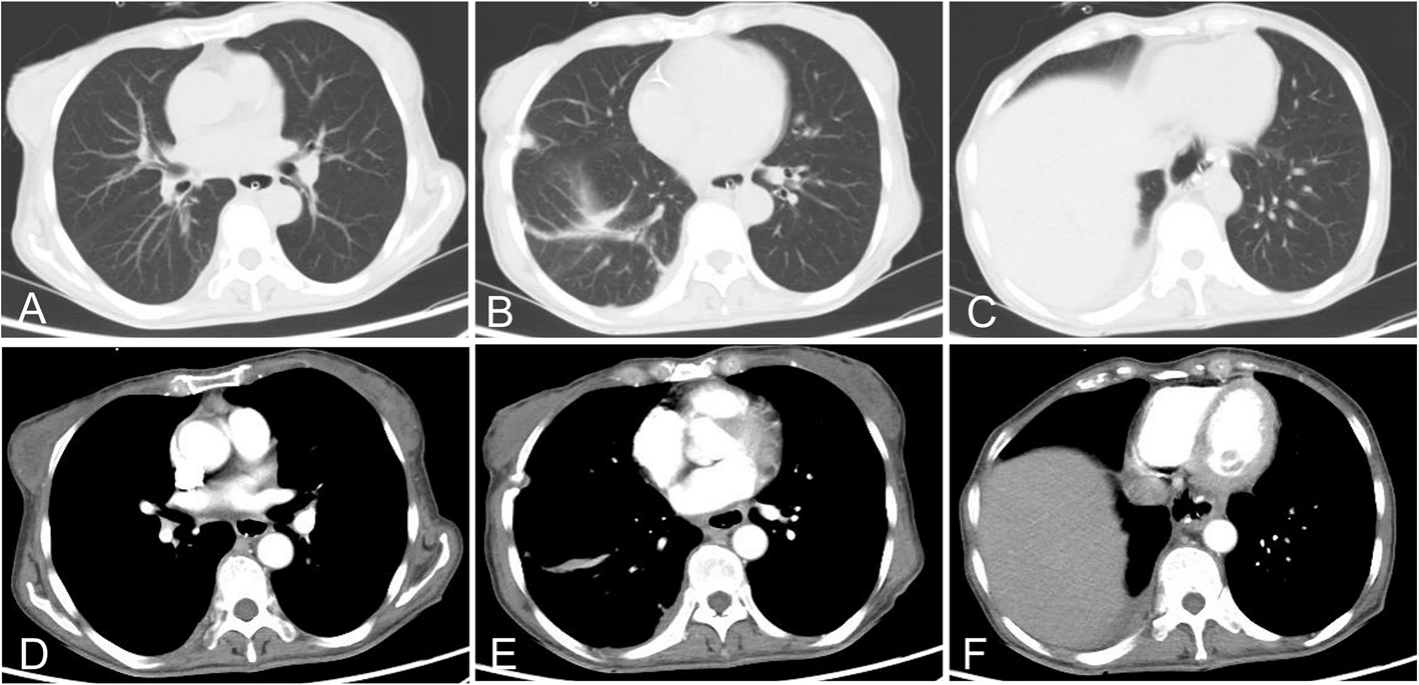
Two months after the intervention, CT scan shows that the accumulated fluid in the lower lobes of both lungs has disappeared, and that the inflammation of the lungs has improved considerably

**Fig. 4:**
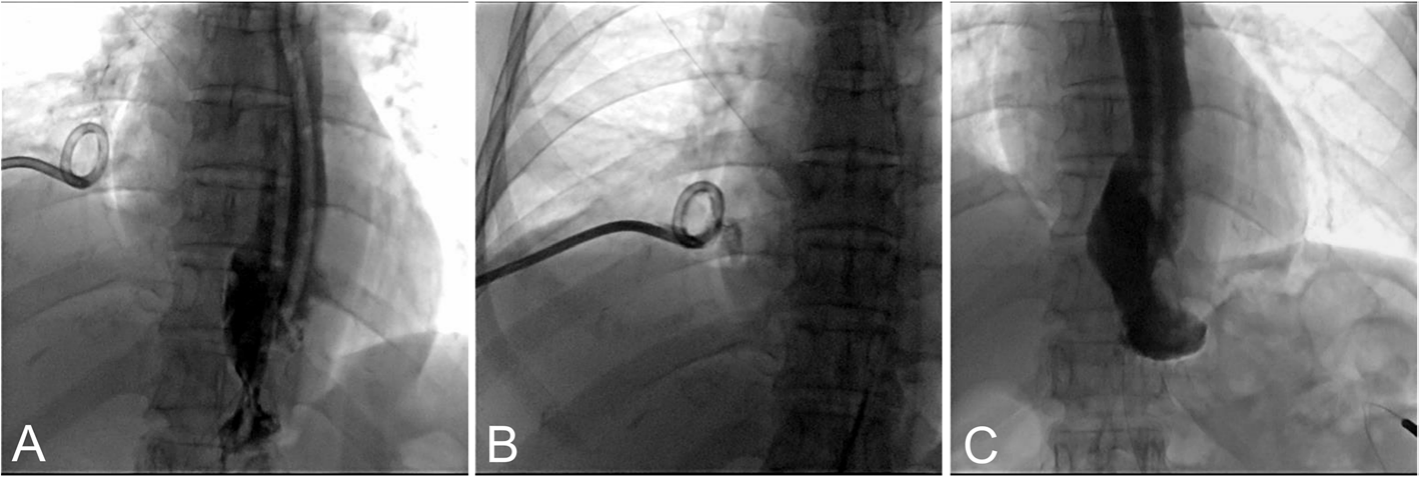
**A** Follow-up esophagography shows smooth passage of contrast through the esophagojejunal anastomosis and no evidence of contrast agent leakage. The fistula has healed, and so the nasal pus drainage tube was removed. **B** Imaging of the percutaneous pus drainage tube shows only sinus tract development, and so percutaneous pus drainage tube was removed. **C** Esophagography after removal of the drainage tube

Esophagography and chest CT were repeated 5–7 days after the procedure to assess abscess cavity size and the efficacy of suction (Fig. 3). When the distal part of the abscess cavity had healed, the position of the drainage tube was adjusted under fluoroscopy so that the tip was at the proximal part. The position of the drainage tube was adjusted every 2 weeks after checking the healing of the abscess cavity; when necessary, the drainage tube was replaced. The percutaneous drainage tube was removed when 5–7 days had passed without any drainage. The transnasal abscess drainage catheter was removed when only a thin line was seen after contrast was injected into the cavity (Fig. 4). The jejunal feeding tube and decompression tube were retained for another week. Oral intake was restarted after esophagography confirmed healing of the fistula.

### Outcomes

Post-procedure quality of life was evaluated by ECOG performance status. Effective treatment was defined as > 50% reduction in the abscess cavity size and improvement in ECOG performance status from that recorded at admission. Treatment success was defined as complete disappearance of the abscess and healing of the fistula, with no recurrence over 6 months of follow-up. Treatment failure was defined as persistence of abscess cavity or fistula, or fistula recurrence within 6 months, need for surgical intervention, or death due to esophagojejunal leak-related complications during treatment. In the literature, the median time to diagnosis of esophagojejunal anastomotic fistula is 8 days [4]; we therefore classified fistulas in this study as early fistula (< 8 days to diagnosis) and late fistula (≥ 8 days to diagnosis).

### Statistical analysis

Statistical analysis was performed using SPSS 26.0 (IBM Corp., Armonk, NY, USA). Continuous variables were expressed as means ± standard deviation. In univariate analysis, variables significantly associated (at *P* < 0.1) with healing of fistula in univariate analysis were included in multivariate Cox regression analysis to identify the independent predictors of healing; hazard ratios and 95% confidence intervals (CIs) were calculated. *P* < 0.05 was considered statistically significant.

## Results

A total of 64 patients (54 males, 10 females; mean age, 63.7 ± 10.3 years) were included in this study (Table 1). R0 resection was achieved in all patients. Postoperative pathology showed lymph node metastasis in 22 patients. Among the 64 patients, 8 received chemotherapy before the surgery, 2 received chemotherapy in the interval between the surgery and the interventional procedure, and 12 patients received intraperitoneal chemohyperthermia with fluorouracil during surgery. Four of the patients had been diagnosed with fistula at other hospitals and had been transferred to our center after failure of endoscopic treatment (1 patient with esophageal stent, 1 patient with titanium clip, and 2 patients with esophageal feeding tube). All four patients were offered the interventional therapy only after further surgical or endoscopic treatments were ruled out by the concerned specialists. Eleven patients had severe infection and respiratory dysfunction and so needed ventilator-assisted ventilation before receiving interventional therapy.

**Table 1:** Characteristics of the study population (*n* = 64)

Characteristic	Data
**Age, years**	63.7 ± 10.3
Sex	
Male	54
Female	10
Comorbidity	
None	29
Hypertension	12
Cancer cachexia	11
Diabetes	9
Coronary heart disease	4
Clinical stage	
Stage I	3
Stage II	14
Stage III	37
Stage IV	4
Unknown	6
Pathological type	
Adenocarcinoma	61
Squamous cell carcinoma	1
Neuroendocrine carcinoma	1
Unknown	1
Differentiation	
Poorly differentiated	21
Moderately differentiation	29
Well differentiated	14
Method of fistula diagnosis	
Esophagography	39
Methylene blue test	12
CT	12
Resurgery for suspected intraperitoneal bleeding	1
Size of fistula, mm	6.95 ± 3.34
Fistula stage	
Early fistula	24
Late fistula	40

Data are *n* or mean ± standard deviation

The most common symptom before the interventional procedure was fever (37/64), and the most common signs were increased drainage and or pus-like changes in the discharge (35/64). The mean white blood cell count before the interventional procedure was 13.4 ± 4.2 × 10^12^/L. The median time from surgery to diagnosis of fistula was 10 days (range, 1–270 days). By the Clavien–Dindo classification, 19 fistulas were type 3, and 45 were type 4. Mean pre-procedure ASA score was 3.9 ± 0.4, mean PSS was 7.9 ± 2.1, and mean ECOG score was 3.4 ± 0.3. The mean time from fistula diagnosis to the interventional procedure was 14.7 days (range, 0–180 days).

Insertion of abscess drainage tube, jejunal decompression tube, and jejunal nutrition tube was successful on the first attempt in all patients. The mean fistula diameter was 6.95 ± 3.34 mm (range, 2.23–16.5 mm). While 35 patients received abscess drainage through the transnasal route, 13 patients received abscess drainage through the percutaneous route, and 16 patients received abscess drainage through both the transnasal and the percutaneous route.

The mean drainage volume was 180 mL (range, 10–850 mL) on day 1; the amount decreased steadily from then on. The mean white blood cell count decreased to 7.9 ± 3.5 × 10^12^/L on day 3 after the interventional procedure. Culture of drainage fluid showed no growth in 18 patients; a single organism was grown in 21 patients and two or more organisms in 25 patients. The most commonly grown organisms were *Escherichia coli* (13/64), *Klebsiella pneumoniae* (9/64), and *Pseudomonas aeruginosa* (9/64). During treatment, there were 31 episodes of blockage of the abscess drainage tube (0–3 times/patient) and 23 episodes of breakage of the abscess drainage tube (0–3 times/patient); displacement, requiring readjustment of the abscess drainage tube position, occurred a mean of 2.7 times per patient (range, 0–11 times).

**Fig. 5:**
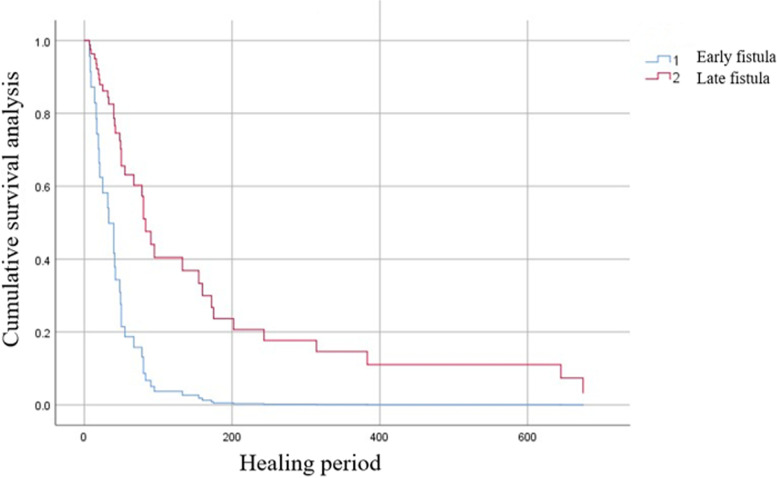
Survival curves of the early and late fistula groups

**Fig. 6:**
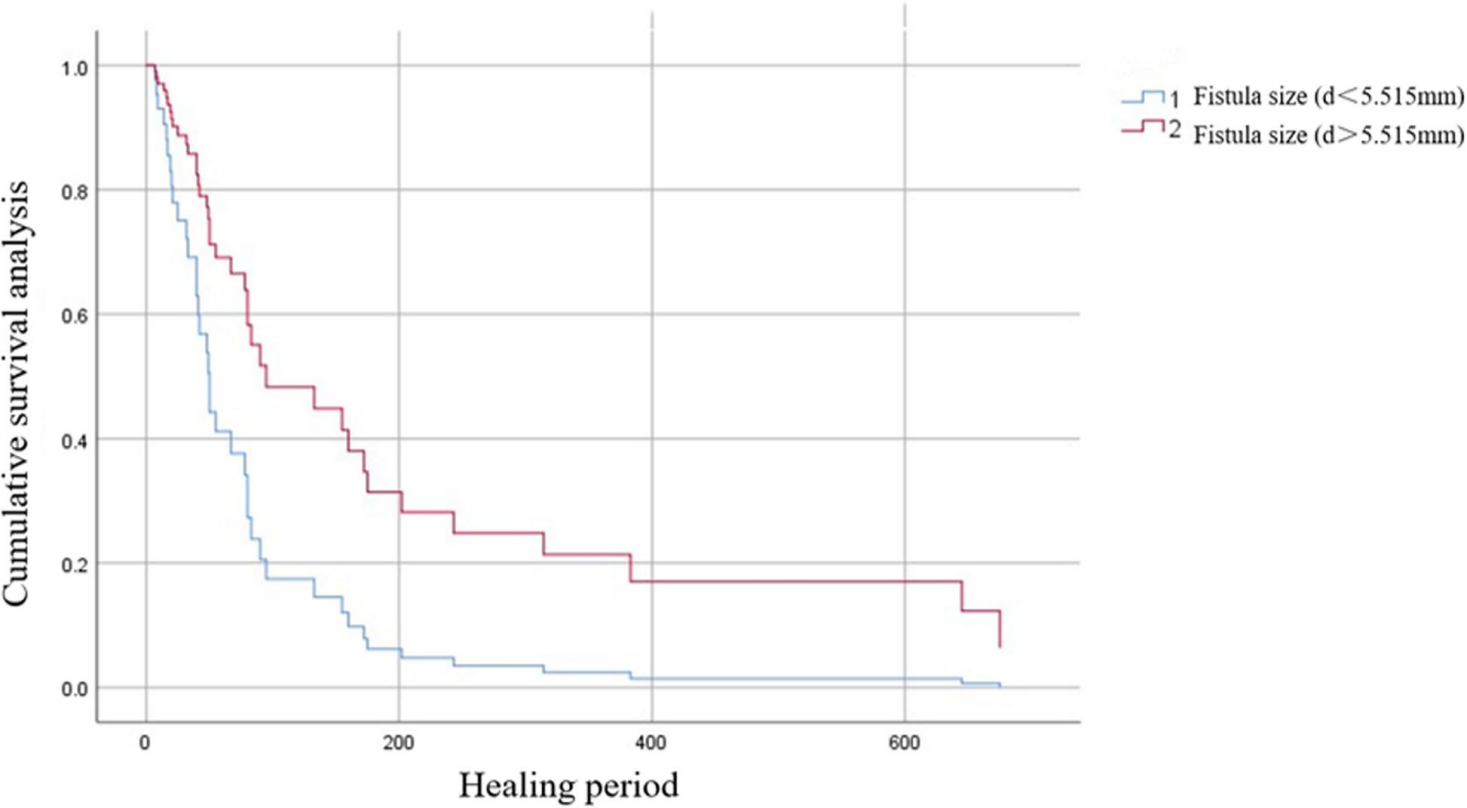
Survival curves of the two fistula size groups

In Cox regression analysis (Table 2), the factors independently associated with healing were fistula stage and size, with late fistulas and large fistulas being predictors of longer healing time (Figs. 5 and 6). Age, sex, preoperative clinical stage, pathological type, complications, and treatment type were not associated with the healing period.

**Table 2:** Predictors of delayed healing of esophagojejunal fistula treated with three-tube method

	Multivariable analysis	
	OR (95% *CI*)	*P*
Age	Not in model	0.601
Sex	Not in model	0.821
Pathological type	Not in model	0.867
Clinical stage (before surgery)	Not in model	0.790
Comorbidity	Not in model	0.398
Fistula stage	3.64 (1.55–8.59)	0.003
Size of fistula	2.40 (1.25–4.60)	0.008
Treatment type	Not in model	0.108

*OR* odds ratio, *CI* confidence interval

### Follow-up

Follow-up data were available for 63 patients until September 2022 (one patient was lost to follow-up). Median follow-up was for 8 months (range, 3–88 months). The most common complications were weight loss (21/63) and reflux (24/63). The fistula healed in 54 patients. The median healing time was 58 days (range, 7–357 days). The mean ECOG score of these patients was 1.9 ± 0.3. A total of nine patients died: four died of tumor recurrence and metastasis 1–7 months after operation, another four died of septic shock or pulmonary infection 0.5–9 months after operation, and one patient died of hematemesis 1 month after operation.

### Complications

During the interventional procedure, there were no cases of hemorrhage, suffocation, or cardiorespiratory arrest. One patient had gastrointestinal hemorrhage 2 days after the procedure, but it was controlled by emergency upper gastroduodenal artery embolization.

## Discussion

Onset of esophagojejunal anastomotic leakage, which generally occurs 5–12 days after surgery, predicts poor outcome. Even with treatment, the mortality rate is in the range of 18–65% [3]. There is still no consensus on the best treatment for esophagojejunal anastomotic fistula; however, the key principles of treatment are isolation of the fistula, adequate drainage, nutritional support, and antibiotic therapy [6, 8].

In this study, transnasal placement of abscess drainage catheter, jejunal decompression tube, and jejunal nutrition tube was performed under local anesthesia in awake patients. The success rate was 100% on the first attempt. We found that the symptoms begin to improve immediately after abscess drainage tube insertion.

In this study, we used two different abscess drainage methods: nasal and percutaneous. We generally prefer nasal drainage because it is non-invasive and simpler to perform; however, when the pus is thick or there is food residue in the pus cavity, we opt for percutaneous drainage. For patients with surgical drainage tubes, we often replace the surgical drainage tube with an interventional drainage catheter because the latter is relatively smaller, easier to carry, and easier to connect to the negative-pressure suction device. The interventional drainage catheter is also more convenient for imaging review to observe the closure of the pus cavity and more easily repositioned when necessary to promote healing of the pus cavity.

Current treatments for esophagojejunal anastomotic leakage include conservative treatment, endoscopic titanium clip, over-the-scope clip (OTSC), esophageal stent, EVT, and surgical treatment. Although these methods are effective, they have limitations. Conservative treatment includes clinical support, infection control with broad-spectrum antibiotics and antifungal treatment, proper drainage, and early nutritional support; however, these measures may not be effective for patients with severe infection [11]. Esophageal stent is associated with many complications, the most common being displacement. Even with use of titanium clips or other measures to fix the stent, displacement occurs in 75–90% of cases [12, 13]. Moreover, occlusion by esophageal stent is not complete, and so treatment effect is uncertain; according to literature, the success rate with esophageal stent is between 44 and 88% [14]. Titanium clips, OTSC, and EVT have been successfully used to treat large fistulas (> 2 cm) and chronic fistulas [1], but they are technically demanding procedures and may sometimes have to be performed under general anesthesia; therefore, they cannot be used in critically ill patients with poor cardiopulmonary function. Although EVT has shown good therapeutic efficacy, with treatment time of 4–78 days [15] and a relatively low complication rate of only 5.56% [14], there are reports of major bleeding during replacement of the sponge. EVT requires change of the sponge every 3–7 days [11, 13]; moreover, the procedure has to be performed under gastroscopy, which is expensive and cumbersome. Secondary surgical repair is a supplementary method for treatment of esophagojejunal anastomotic leakage. It is indicated in patients who have failed endoscopic treatment or in those with severe complications such as intra-abdominal hemorrhage, sepsis, and giant anastomotic leakage; however, surgical repair is unsuitable for patients who have had recent surgery and have hypoproteinemia or for those with sepsis and unstable blood pressure [16]. The mortality rate in patients undergoing reoperation is 65–75% [16, 17].

Bachmann et al. classified anastomotic fistula by severity according to endoscopic findings and devised corresponding treatment measures [18]. Endoscopic evaluation of fistula condition is valuable but used gastrointestinal imaging under fluoroscopy to identify contrast agent leakage and formation of pus cavity. Persistence of pus in the cavity makes repair or sealing of fistula difficult. We believe that promoting healing of the pus cavity should take precedence over the closure of the fistula. During the treatment process, when gastrointestinal imaging shows no evidence of contrast agent leakage, we perform gastroscopy to evaluate fistula healing. In previous studies, we have demonstrated that the three-tube method is effective and feasible for treatment of duodenal fistula [19]. We have found that as long as the three-tube placement is accurate, suction is effective, and nutrition is guaranteed, rapid improvement in general condition of the patient and ultimate healing of the pus cavity and fistula can be achieved.

Our method of nasal insertion of abscess drainage catheter, jejunal decompression tube, and jejunal nutrition tube for treatment of esophageal jejunostomy fistula has several advantages. First, it can be performed under local anesthesia in the awake state and so is suitable for critically ill patients with poor cardiopulmonary function. Second, placement of the tubes can be accurately performed under fluoroscopy. Third, the application of continuous suction prevents accumulation of pus and creates a negative pressure environment that is conducive to the healing of the abscess cavity. A negative-pressure environment is conducive to extracellular matrix remodeling and granulation tissue deposition and, thereby, closure of the pus cavity [20, 21]. In theory, it reduces the probability of gastrointestinal bleeding caused by the erosion of arteries in the abscess wall. Fourth, regular esophagography and fistulography through the drainage tube can be performed and the position of the drainage tube adjusted to ensure effective drainage. When necessary, the drainage tube can easily be replaced by the interventional technique under fluoroscopy. Fifth, the procedure cost is low compared with EVT under endoscopy, as EVT requires 3–8 sponge replacements per person per session. Finally, drainage is an established method for treatment of abscesses, and interventional devices are relatively safe. There are reports that the misplacement and/or long-term placement of drains close to the greater curvature of the stomach can cause perforation of the stomach wall [22]; in comparison, the catheter guide wire used in our method is very flexible, and its introduction under fluoroscopy is minimally traumatic. So far, there has been no report of bowel perforation resulting from use of interventional devices.

For successful interventional treatment, the intervention should be gentle and precise. Imaging should be performed after placement of the drainage tube to confirm correct positioning of the tube; easy aspiration of contrast agent is proof that the drainage tube is in the right position. Regular imaging of the abscess cavity, with adjustment of the position of the drainage tube when necessary, can accelerate healing. Regular tube care is necessary. We have found that breakage and blockage of the drainage tube are common. This may be because we presently use an angiography catheter as the drainage tube; we hope that a dedicated transnasal abscess drainage tube will become available soon.

The study has limitations. First, it is a single-center retrospective study, and so a selection bias is inevitable. Second, the sample size is relatively small. Third, there was no control group.

In conclusion, for gastric cancer patients with esophagojejunal anastomotic fistula after gastrectomy, interventional treatment under fluoroscopy, with transnasal insertion of abscess drainage tube, decompression tube, and jejunal nutrition tube, appears to be a feasible, safe, and effective treatment. The interventional three-tube method is a promising new approach for treatment of esophagojejunal anastomotic fistula. This simple and minimally traumatic procedure can also be performed in very ill patients. Large multicenter controlled studies are needed to confirm our findings.

## Data Availability

The datasets used and/or analyzed during the current study are available from the corresponding author on reasonable request.

## References

[1] Wang W, Qi K, Chang X, Jin Z, Li Z (2019). Two-session endoscopic purse-string suture to close a huge esophagojejunal anastomosis thoracic cavity fistula. Endoscopy.

[2] Trapani R, Rausei S, Reddavid R, Degiuli M. Clinical investigators. Risk factors for esophago-jejunal anastomosis leakage after total gastrectomy for cancer. A multicenter retrospective study of the Italian research group for gastric cancer. Eur J Surg Oncol. 2020. 10.1016/j.ejso.2020.06.035. 10.1016/j.ejso.2020.06.035 32703713

[3] Makuuchi R, Irino T, Tanizawa Y, Bando E, Kawamura T, Terashima M (2019). Esophagojejunal anastomotic leakage following gastrectomy for gastric cancer. Surg Today.

[4] Barchi LC, Ramos MFKP, Pereira MA (2019). Esophagojejunal anastomotic fistula: a major issue after radical total gastrectomy. Updates Surg.

[5] Xing J, Liu M, Qi X (2021). Risk factors for esophagojejunal anastomotic leakage after curative total gastrectomy combined with D2 lymph node dissection for gastric cancer. J Int Med Res.

[6] Oka S, Sakuramoto S, Chuman M, Aratani K, Wakata M, Miyawaki Y (2017). Successful treatment of refractory complete separation of an esophagojejunal anastomosis after laparoscopic total gastrectomy: a case report. BMC Res Notes.

[7] Jung GM, Lee SH, Myung DS, Lee WS, Joo YE, Jung MR (2018). Novel endoscopic stent for anastomotic leaks after total gastrectomy using an anchoring thread and fully covering thick membrane: prevention of embedding and migration. J Gastric Cancer.

[8] Hallit R, Calmels M, Chaput U, et al. Endoscopic management of anastomotic leak after esophageal or gastric resection for malignancy: a multicenter experience. Therap Adv Gastroenterol. 2021. 10.1177/17562848211032823. Published 2021 Jul 23. 10.1177/17562848211032823 PMC8832292 35154387

[9] Wu G, Zhao YS, Fang Y, Qi Y, Li X, Jiao D (2017). Treatment of spontaneous esophageal rupture with transnasal thoracic drainage and temporary esophageal stent and jejunal feeding tube placement. J Trauma Acute Care Surg.

[10] Wu G, Zeng YW, Wang JX, Ma W, Yin MP, Zhao Y (2020). An interventional radiology technique to treat pharyngeal or esophageal perforation associated with mediastinal abscess in children. J Pediatr Surg.

[11] de Moura DTH, de Moura BFBH, Manfredi MA, Hathorn KE, Bazarbashi AN, Ribeiro IB, et al, Thompson CC. Role of endoscopic vacuum therapy in the management of gastrointestinal transmural defects. World J Gastrointest Endosc. 2019. 10.4253/wjge.v11.i5.329. 10.4253/wjge.v11.i5.329 PMC6556487 31205594

[12] Emre A, Sertkaya M, Akbulut S, et al. Self-expandable metallic stent application for the management of upper gastrointestinal tract disease. Turk J Surg. 2018. 10.5152/turkjsurg.2017.3740. Published 2018 Apr 30. 10.5152/turkjsurg.2017.3740 PMC6048644 30023972

[13] Kim HS, Kim Y, Han JH (2021). Endoscopic salvage treatment of histoacryl after stent application on the anastomotic leak after gastrectomy: a case report. World J Clin Cases.

[14] Rausa E, Asti E, Aiolfi A, Bianco F, Bonitta G, Bonavina L (2018). Comparison of endoscopic vacuum therapy versus endoscopic stenting for esophageal leaks: systematic review and meta-analysis. Dis Esophagus.

[15] Schorsch T, Müller C, Loske G (2013). Endoscopic vacuum therapy of anastomotic leakage and iatrogenic perforation in the esophagus. Surg Endosc.

[16] Seesing MFJ, Gisbertz SS, Goense L (2017). A propensity score matched analysis of open versus minimally invasive transthoracic esophagectomy in the Netherlands. Ann Surg.

[17] Carboni F, Valle M, Federici O (2016). Esophagojejunal anastomosis leakage after total gastrectomy for esophagogastric junction adenocarcinoma: options of treatment. J Gastrointest Oncol.

[18] Bachmann J, Feith M, Schlag C, Abdelhafez M, Martignoni ME, Friess H (2022). Anastomotic leakage following resection of the esophagus-introduction of an endoscopic grading system. World J Surg Oncol.

[19] Wang S, Han XW, Wu G (2022). A new technique for the treatment of duodenal fistula. Asian J Surg.

[20] Glass GE, Murphy GF, Esmaeili A, Lai LM, Nanchahal J (2014). Systematic review of molecular mechanism of action of negative-pressure wound therapy. Br J Surg.

[21] Wichmann D, Stüker D, Schweizer U, et al. Endoscopic negative pressure therapy for duodenal leaks. Front Surg. 2023. 10.3389/fsurg.2023.1099457. Published 2023 Apr 18. 10.3389/fsurg.2023.1099457 PMC10151564 37143771

[22] Shao HJ, Lu BC, Xu HJ, Ruan XX, Yin JS, Shen ZH (2016). Gastric fistula secondary to drainage tube penetration: a report of a rare case. Oncol Lett.

